# Critical assessment of relevant methods in the field of biosensors with direct optical detection based on fibers and waveguides using plasmonic, resonance, and interference effects

**DOI:** 10.1007/s00216-020-02581-0

**Published:** 2020-04-20

**Authors:** Günter Gauglitz

**Affiliations:** Institute of Physical and Theoretical Chemistry, Eberhard Karls Universität, Auf der Morgenstelle 18, 72076 Tübingen, Germany

**Keywords:** Biosensor, Evanescent field, Resonance, Refractometry, Reflectometry, Interference

## Abstract

Direct optical detection has proven to be a highly interesting tool in biomolecular interaction analysis to be used in drug discovery, ligand/receptor interactions, environmental analysis, clinical diagnostics, screening of large data volumes in immunology, cancer therapy, or personalized medicine. In this review, the fundamental optical principles and applications are reviewed. Devices are based on concepts such as refractometry, evanescent field, waveguides modes, reflectometry, resonance and/or interference. They are realized in ring resonators; prism couplers; surface plasmon resonance; resonant mirror; Bragg grating; grating couplers; photonic crystals, Mach-Zehnder, Young, Hartman interferometers; backscattering; ellipsometry; or reflectance interferometry. The physical theories of various optical principles have already been reviewed in detail elsewhere and are therefore only cited. This review provides an overall survey on the application of these methods in direct optical biosensing. The “historical” development of the main principles is given to understand the various, and sometimes only slightly modified variations published as “new” methods or the use of a new acronym and commercialization by different companies. Improvement of optics is only one way to increase the quality of biosensors. Additional essential aspects are the surface modification of transducers, immobilization strategies, selection of recognition elements, the influence of non-specific interaction, selectivity, and sensitivity. Furthermore, papers use for reporting minimal amounts of detectable analyte terms such as value of mass, moles, grams, or mol/L which are difficult to compare. Both these essential aspects (i.e., biochemistry and the presentation of LOD values) can be discussed only in brief (but references are provided) in order to prevent the paper from becoming too long. The review will concentrate on a comparison of the optical methods, their application, and the resulting bioanalytical quality.

## Introduction

The measurement of molecule interactions in medicine, biology, biochemistry, and diagnostics has been of high importance. Many years ago, radio-labelling has been used to report the binding of a ligand to its receptor. One analytical development-pushing application has been drug discovery [1] to determine affinity, activity, toxicity or availability of candidates in the process of ligand/receptor interactions. Especially screening applications have driven research beyond ELISAs to receive thermodynamic as well as kinetic data in biomolecular interaction analysis (BIA) [2–4]. Primary screening of antibodies and selection of alternative binders out of cell cultures at extremely low concentrations with high throughput are present high topics. In the future, screening of large data volumes will get interest in immunology and cancer therapy. This aims to personalize medicine, and methods without labelling will be of interest in **C**lustered **R**egularly **I**nterspaced **S**hort **P**alindromic **R**epeats genome editing (CRISPR/Cas) methods in molecular biology [5].

The huge field of biomolecular interaction analysis and its application to urgent problems in the environment, biology, medicine, and health care has induced an extreme number of publications in classical analytics like MS, NMR, or hyphenated techniques with separation science as well as reporting biosensors based on mass-sensitive, electronic, electrochemical, or optical devices. The scope of this review is to try to name the problems using biosensors coming from biochemistry, surface chemistry, transport processes in sample cells, microfluidics, and detection. For space reasons, not all problems can be discussed in detail. Therefore, after a brief survey of potential sensor principles, the review has to focus on optical detection and especially on direct optical detection. Thereby, the physical and optical basics will be referenced and not discussed using formulas. The main aim is to classify these optical principles according to plasmonic, resonance, or interferometric effects to give the reader a systematic picture of the huge number of devices, many with just minimal modification of the original optical principle. For this reason, also the “historical” development is sketched in most methods. In Chapter conclusion, Table 1 shows the essential parameters.

## Biomolecular interaction analysis

A successful approach to achieve information without radio-labelling is isothermal titration calorimetry (ITC) which yields thermodynamic data such as enthalpy of binding or entropy of binding of especially large biomolecules. Typically, protein/protein interactions are examined [68]. The application of ITC for the formation or disassociation of molecular complexes has developed since first publications in 1990 [69]. Since that year, the number of publications has increased, and publications cover especially the field of protein chemistry. Research and technical development from 2011 to 2015 has been reviewed, providing information on methodological advances and interpretation of single and multiple binding sites [70]. Besides thermodynamic information on binding constants, e.g., enzymes, of substrate reactions and inhibitory constants, kinetic data is also of interest. The possibilities are introduced in [71]. Modern ITC instrumentation allows measurement of very small heat powers and provides a tool for biology to study association processes involving liquid membrane proteins, nucleic acids, macromolecular assemblies, and a great variety of ligands. A joint method for thermodynamic and kinetic data achieved by ITC is described in [72]. Miniaturized calorimeter with an elaborated temperature control inside the system was developed for microbiological applications [73]. Despite the instrumental and methodological development, the ITC is a calorimetry not easy to handle and lacks screening possibilities. Thus, one could realize increasing interest to have another method to determine thermodynamic and kinetic data of the biomolecular interaction process.

Since approx. the year 2000, biosensors as a tool for quantifying ligand/receptor interactions in homogeneous phase and at heterogeneous interface came more and more into focus. There is a huge variety of biosensor types, ranging from mass-sensitive (quartz-microbalances (QCM), surface acoustic waves (SAW), or cantilever (CL) systems) to electrochemical and optical ones. Recently, a survey on sensors in general and their application has been published [74, 75]. All these possible biosensors depend on sampling, sample preparation, suitability for microfluidics setups, potential parallelization, and miniaturization and finally in the case of direct detection without label on avoiding rival non-specific binding in the biomolecular interaction process. Because of these many requirements considering biosensing, first, a brief survey on non-optical method is given with reference to quality and applicability to problems in biochemistry, biology or medicine, just to demonstrate the huge variety of methods.

QCM, SAW [76], and CL [77, 78] based sensors are especially suitable to monitor mass-sensitive effects. Therefore, their applications are well known for measuring gas concentrations. However, the signal depends on liquids extremely on viscosity. The Sauerbrey equation is not anymore applicable in its simple form. Interfacial properties of solid-liquid interface have to be considered [79]. Nevertheless, QCMs are used for biosensing [80, 81] and even cell behavior is examined [82]. Picomolar specific biomarker target detection was achieved for miss-matches of non-coding RNA [83] or using micro-cantilever arrays for early liver cancer diagnosis [84]. However, the mechanical sensitivity to damage prevents out-of-lab applications. For many years, electrochemical sensors were preferably used in biosensing. Especially the possibility to fabricate large sensor arrays with many spots or readers for microtiter plates supported many applications [85, 86]. The development of impedance instrumentation, which allowed parallel measurement of signal and phase, pushed electrochemical biosensors [87, 88]. Applications of DNA-based electrochemical sensors are numerous [89]. Recently the literature on bioanalytics using microelectrodes has been reviewed [90]. A smart and interesting combination of electrochemistry and chemiluminescence results in many advantages such as remarkably lower limits of detection, higher sensitivity, and a wide dynamic range. This electrochemiluminescence (ECL) [91] offers many applications like measuring toxins, pesticides or drugs in food [92]. It has improved with new graphene electrodes [93]. Comparable with electrochemical biosensors a huge amount of publications deals with detection principles and applications of optical biosensors. A very detailed review on optical biosensors provide — apart from the definition of biosensors — descriptions of different recognition elements as enzymatic biosensors, immunosensor, ligand/receptor interactions, and nucleic acid assay and even whole cells. Furthermore, the paper provides an in-depth survey on methods and applications [94]. Recently, a review on biological and synthetic materials as recognition elements for food safety analysis has been published [95, 96] can help to select a suitable recognition element. Because of the wide field of detection principles and applications the review aims to focus on optical biosensing and within these many methods is restricted to biosensors based on direct optical detection.

## Optical biosensors

### General considerations

Of the optical biosensors, at first, fluorescence assays (using a marker or a label) were predominantly used, taking into account that problems with photostability and influences on the bioactivity of fluorophore-labeled partners could prevent to obtain kinetic data apart from equilibrium constants. However, regarding the possibility of measuring multiple interactions in parallel, microarrays based on methods using labels proved their advantages [97, 98]. The detection of biomolecular interaction in bulk or homogeneous phase on top of the transducer is only possible with signal changes caused by variation of the fluorescence intensity or change of fluorescence wavelength of the monitored complex. In addition, the quenching of fluorescence might be used during the interaction process. However, this quenching also can be caused by simple changes in the oxygen concentration of the solution, for example. Therefore, this effect is rather non-specific. A better chance offers (Förster) fluorescence resonance energy transfer (FRET) where the ligand as a labeled donor molecule and a fluorescent receptor interact. The receptor absorbs at the fluorescence wavelength of the receptor in the case of a close neighborhood (up to 10 nm) by dipole interaction. Fluorescence intensity of the receptor increases [99, 100]. However, even this effect depends often on environmental conditions. Reviews on fluorescence biosensors have been published some years ago [101–105]. Another possibility in the homogeneous phase is the measurement of the light scattering which will differ dependent on the size of the complex measured. However, in this case, the interaction between a large receptor and a small ligand will give a poor variation of the signal. Only interactions with recognizable differences in size between ligand/receptor and the complex can be monitored [106, 107]. Accordingly, normally in optical biosensing, a heterogeneous phase device is used.

Soon, after 2000, the advantages of direct optical detection were discussed, especially in the case of drug discovery technologies [108]. Rather early upcoming new interesting field were considered in which direct optical sensing promised advantages [109]. Faster assay development times, accurate and high information content data, and less interference from labels were considered as an advantage and as a perspective [110]. In the following, a large number of optical devices were developed. An early review about the many possible label-free biosensor structures also exists [111]. The principles of these optical methods are discussed and compared in [112]. Progress in material fabrication and novel substrate with enhanced optical response properties and potential application for rapid analytical measurement of target interactions from proteins to DNA and viruses are demonstrated in a review article on emerging applications [113]. Looking especially at small molecules, many techniques, including surface-enhanced Raman spectroscopy, have been reviewed in a recent article, together with potential evaluation techniques [114]. Raman and especially using the SERS (surface-enhanced Raman spectroscopy) instrumentation has become very interesting because of new developments resulting in easy to use and at low costs [115]. Nevertheless, Raman cannot be the topic of this review.

The direct optical detection techniques perform spectroscopy on biomolecules at the surface of the transducer. Accordingly, the measurement not only depends on the transduction method but also on competition between specific and non-specific interactions. Labelling can reduce the problems with non-specific interaction, but in the case of direct optical detection, this problem always arises [116]. Therefore, normally between the transducer and the recognition sites (responsible for the amount of biomolecular interaction) a biolayer is added, which reduces and/or prevents non-specific binding and allows the immobilization of as many recognition sites as possible [117]. Besides, nonspecific interaction, the performance of direct optical sensors is impaired for very small analytes, which do not provoke recognizable signal changes when interacting with recognition elements at the sensor surface. This problem can be overcome by using either competitive or binding inhibition assay formats [118].

In general, the optical techniques, which will be discussed here, use the influence on the propagation of electromagnetic radiation in a waveguide or fiber or effects on the reflection of electromagnetic radiation at the interface including resonance and interference effects. In principle, all direct optical detection techniques measure the product (*n* × *d*) of refractive index n and the physical thickness of an interaction layer d. Depending on the transduction method used and on the setup of the measurement cell, the readout is dominated by the influences on the refractive index during bio-recognition or on the changes of the physical thickness of the examined layer during interaction. Thus, it is possible to divide direct optical detection techniques into refractometric- and reflectometric-based fundamentals. In the case of refractometric dominance, the so-called evanescent field outside the waveguide is influenced by the optical density on top of this waveguide (mostly by the refractive index) [119–121]). Whereas interferometric methods monitor changes in the interaction layer homogeneously across the total radiation pathway, the refractive index exponentially decays with distance to the waveguide transducer within a few hundreds of nanometers. Thus, large shielding layers and/or large interacting molecules (cells) cause problems (see LRSPR). Furthermore, the temperature dependence of refractive index should be considered in referencing.

The main aim of this review is a survey on resonance and interferometric methods used presently in biosensing, with some trends in recent literature. Especially the waveguide-based optical methods predominantly rely on changes of the refractive index in the sample. Among the interferometric methods, some rely on evanescent field techniques, which combine refractometry with interference; some use resonator systems which also include interferometry; and finally, the typical interference reflectometric methods have to be mentioned. In total, an extremely large number of realization of the basic optical principles exist. Sometimes modifications are rather small and specific. The quality of the method regarding the limit of detection, reproducibility, or sensitivity depends on the application in many publications. Thus, a comparison is sometimes difficult just considering the optical transduction principle. Furthermore, besides the kinetics at the recognition sites, the mass transport from the bulk to the recognition sites plays an interesting role in dependence on the loading with recognition sites [122, 123]. Thus, aspects of biomolecular interaction analysis have to be considered [2], understanding the ratio of transport limited interaction to kinetics at the surface in dependence on the loading of the surface with recognition elements versus concentration of ligand in the homogeneous phase [3, 124]. These aspects will be more considered in interferometric applications.

### Fiber- or waveguide-based biosensors

In optic communication the fibers find wide usage to transport electromagnetic radiation between the two ends of the fiber to transmit the incident radiation of a light source to a detector. Besides this usage in biosensor applications, another property of fibers is used. The inside radiation pathway is determined by total internal reflection. Thus, fibers act as a waveguide. Because of quantum optics to this electric field vector an external electric field vector couples forming an evanescent field outside into the bulk (cladding or sample) close to the core of the fiber/waveguide. Whereas in fibers the core is surrounded by a transparent cladding, for waveguides higher refractive index material is structured onto a substrate and covered by a thin layer or contacts directly the sample; both having lower refractive index values. Both the guided wave (at total reflectance conditions) and the resulting evanescent field depend on the core of the fiber/waveguide and on the cladding/substrate. The theory of waveguiding is discussed in many textbooks and elsewhere [119, 125]. Any influence on the refractive index within this evanescent field will influence the guided wave, since the electric field of the evanescent wave couples back to the electric field vector of the guided wave and result in an effective refractive index. The principle of such a transducer is to find possibilities to readout this effective refractive index and its changes by activities close to the waveguide. Different types of structures of waveguides such as slap, buried, diffused, strip-loaded, ridge, rib, or even ARROW waveguides are discussed in principle in [126]. A survey on various realizations, especially with a view to the influence on the two modes TM and TE (transversal magnetic/electric) of electromagnetic radiation in the waveguiding optics, is given in [127].

In summary, in a fiber or waveguide, radiation propagates via total internal reflectance. For the following discussion of optical sensor principles, the general questions are as follows: (1) How can radiation be brought into the fiber/waveguide to propagate via total internal reflectance? (2) Which external effects influence this internal propagation? and (3) How does the readout of the influence on this propagation of radiation work? Therefore, for such types of transducers the following points have to be considered: the in-coupling of radiation (angle, wavelength, state of polarization), the properties of the generated evanescent field, and for the readout the achieved intensity, out-coupling angle, wavelength, state of polarization, change in phase of the radiation. Classification according to waveguide, resonance, or interference is rather difficult, since many of the methods use optics relying on different methods, sometimes in combination.

Essential is the in-coupling of electromagnetic radiation into the waveguide which results in total reflectance conditions of the guided wave and reducing losses during in-coupling. In-coupling can be achieved via a lens as an end-fire coupling, or simply by butt-end coupling, or via a prism or a grating or even by using the coupling of two waveguides via their interacting evanescent fields. The modes of the guided wave may differently depend on the value of the refractive index of the core and the surrounding of the waveguide, on the material, and on the influence of external refractive index changes via the evanescent fields on the guided wave (optically isotropic/anisotropic). Accordingly, the phase of the modes can depend on the diameter dimensions of the waveguide, forming mono-mode or multi-mode propagation. If the two modes have different propagation conditions, a phase shift between both will occur. Internal and external structuring of the waveguide is possible, will influence the phase conditions of the modes, and may cause resonant and interference conditions inside the waveguide. Accordingly, a very large number of possible readout realizations can be found in literature for direct optical detection; however, it should be kept in mind that besides all realizations of optical theory, the quality of a biosensor is certainly application-driven and depends to a large degree on the quality of the biochemistry in order to obtain an optimized biosensor. In the following the main approaches for in-coupling of radiation, to influence the propagation in the fiber/waveguide, and for readout information are discussed.

#### Fibers and waveguides without structure

The interaction of the evanescent field coupled to the guided wave internally a fiber or a waveguide was used for sensing already at an early time (EFAS: evanescent field absorbance sensor). Attenuation of the waveguide could be measured if the interaction distance was long [128]. Improvement also for measurements in the NIR was achieved by long path integrated optical sensor chips [129]. These realizations of a fiber/waveguide sensor were not followed in the future because of mechanical instability and coupling problems with the micro-chips. Thus, future research concentrated on fiber/waveguide modifications and methods of better readout.

##### Coated fibers

A possibility is the use of optical fiber sensing based on Brillouin scattering with different approaches such as Brillouin optical frequency correlation domain analysis or even correlation domain reflectometry [130]. Radiation interacts with the material waves in a medium in dependence on the material properties and is (back-)scattered by periodic fluctuations of phonons. These can be influenced by temperature or strain. Accordingly, the elastic behavior of thin films can be measured (potential application in garments). Various approaches of optical frequency domain reflectometry are reviewed in [6]. The latter systems are rather complex and are not yet applied widely. They might become interesting application in fibers imbedded in clothing.

There is a large variety of realizations of optical fibers or waveguides with polymer cladding or metal clads. Details are discussed elsewhere. The possibility to use metal cladding on fibers or waveguides for chemo- or biosensing had been introduced rather early [7]. Waveguides coated with a thin gold layer and a buffer layer between the waveguide and the metal film offered the chance of surface plasmon resonance (SPR) [131]. This buffer layer is necessary to reach the in-coupling condition for total internal reflectance. Various types of metal clad waveguides had been compared [132]. Label-free biosensing platforms based on planar optical waveguides have been discussed with respect to their operation principles and performance characteristics [133], four devices for generating SPR using optical fiber are compared for biosensor applications [134]. A survey on modifications of optical fibers and applications in various fields —especially diagnostics — in combination with a discussion of future challenges are given in [135]. Nanowires, nanoparticles, and nanoholes are used for biosensing [136]. A large number of types of fiber sensors are reviewed [137] and challenges and prospects are discussed recently [138]. Nanoparticles are used directly for detection also for signal enhancement. Their applications in various detection methods are compared in the case of gold nanoparticles recently [8].

##### Ring resonator

The signal is restricted in all mentioned instrumental developments by the interaction length. Therefore, ring resonator systems were considered to overcome these limitations. In part of [111] an extensive review is given. At total reflectance conditions, electromagnetic radiation travels in a ring micro-waveguide in substrate and the evanescent field forms the so-called whispering gallery modes characterized by a number of wavelengths in this orbit. Extremely sensitive to waveguide and outside refractive index, a resonator with a quality factor is formed. Based on the first experiments [139], soon, the first application to biosensing was published [140, 141]. The first years of development and application as well as some configurations are given in [9]. Recent activities are demonstrated in [142]. In many publications, the high sensitivity is argued as an advantage of such ring resonator systems [143]. In [10], optical biosensors based on integrated photonic devices with a special view on silicon-on-insulator ring resonators are reviewed with respect to sensing mechanism, sensor design, and biofunctionalization. Even a high-quality factor (low loss within the ring) with a detectable small wavelength shift cannot compensate for the small coupling area and biomolecular interaction conditions. Thus, the presented limits of detection (interleukin 6–100 pM) are comparable with other direct optical sensors. These scalable and cost-effective on-chip biosensors can be interesting for a broad market in the future. In [144], biosensors based on silicon photonics (among ring resonators) are compared with respect to chip-scale integration and miniaturization with potential for low-cost, high yield and portability in applications also for point-of-care diagnosis.

##### Difference interferometer

In 1991, the term difference interferometer was introduced as a new type of integrated optical interferometer, using a mono-mode SiO_2_-TiO_2_ waveguide in which the TE and TM modes are coherently excited. The time-dependent phase difference is measured in dependence on the interaction of the waveguide with a sample. The properties of this difference interferometer as a differential refractometer were applied first as a humidity sensor [145], and later for applications as a biochemical sensor, beginning with monitoring avidin-biotin-BSA affinity reactions [146]. Avidin and biotin have a very high equilibrium constant and are easily determined even at lower concentrations. Many publications use this equilibrium as a first test for biomolecular interaction and give nice limits of detection which are not at all attainable with relevant analytical problems. Thus, concentrations of 50 μg/L biotin BSA could be detected on streptavidin layers. The use of Wollaston prisms in this difference interferometer was to separate TE and TM mode propagation, and the theoretical background is given in [147, 148]. The difference interferometer was also applied to direct affinity sensor measurements. Limits of detection of anti-h-IgG with 10^−11^ M are achieved because of the high molecular weight of more than 100 KDa. In the following years, the complex readout by Wollaston prism was complemented by the interference of out-coupled modes TE and TM forming interference fringes with a polarizer from the surface-relief grating [11]. Another approach was a dual-wavelength difference interferometer [149], in which end-fire coupling with Wollaston and polarizer form time-dependent spatial interference fringes which are recorded by a CCD. Additional advantage is a dual-wavelength operation which allows the separation of surface-mass-density changes and sample’s refractive index changes or temperature fluctuations. Readout of difference interferometers is the phase difference of the two modes. The dependency of the modes in the case of polymer coatings was simulated and measured, even for a multilayer system being a bimodal waveguide [150]. Sensitivity and selectivity of this difference interferometry is discussed in comparison with SPR and input grating couplers [12]. A monolayer coverage for IgG-complex is determined to 5 × 10^−9^ g mm^−2^.

##### Surface plasmon resonance

In a high percentage of research articles describing direct optical detection for biomolecular interaction processes, surface plasmon resonance (SPR) is used in various modifications as a successful tool. SPR was first introduced to biosensing and gas detection in 1983 [151]. Electromagnetic radiation is in-coupled by a prism at total internal reflection conditions. The prism is coated with a thin gold film of approx. 50 nm. At resonance conditions (suitable wavelength and / or angle of incidence of radiation) the TM mode (transverse magnetic mode propagation [119]) excites surface plasmons in the metal film (near the metal surface) and forms an evanescent field, reaching into the volume close to the surface of the metal film (opposite interface to the incident one at interface metal/prism). The intensity of the reflected electromagnetic radiation is reduced under resonance conditions, and a “dip” is formed in the “reflection spectrum” [119, 152]. This type of “waveguide” based sensor has been also named as a prism coupler [153].

Any change in the refractive index in the sample cell close to the interface of the metal film varies the resonance condition and therefore the position of the dip in the “spectrum.” Since this method was commercialized at an already early time (https://www.gelifesciences.com/en/gb/solutions/protein-research/products-and-technologies/spr-systems, https://www.gelifesciences.com/en/us/solutions/Protein-Research/Knowledge-center/Surface-plasmon-resonance/Surface-plasmon-resonance), there exist a large number of publications covering the application of this method (originally pSPR: propagating SPR). A large number of these applications were described in reviews [154]. pSPR as a normal approach uses a thin metal film. The localized SPR (lSPR) uses nanoparticles on a glass layer. The pSPR setup shows an influence on the amount of reflectance, whereas the lSPR is usually measured in transmittance. Both approaches are compared with each other based on theoretical calculations and experiments [155]. The pSPR system is significantly better compared with the lSPR with regard to the measurement of the bulk refractive index. However, lSPR improves the measurement of small molecules when smaller nanoparticles are used (signal depends on the nanoparticle size). Among SPR used for biosensing, there are four typical types: the conventional pSPR, the long-range SPR (LRSPR), the classical plasmon-waveguide resonance (CPWR) and the waveguide-coupled SPR (WCSPR). All these rely on attenuated total reflection; their sensitivities are compared in [156]. Recently, the fundamentals and upcoming technological advances and their applications have been discussed [15, 157], even in comparison with other direct optical sensors. SPR has become a gold standard for biomedical diagnostics including point-of-care diagnostics. SPR sensing of nucleic acids was reviewed [16], demonstrating the concept of such SPR biosensors in case of nucleic acid detection, the immobilization techniques, fabrication of arrays and quantification strategies in medical diagnostics [158], food safety [159], and environmental monitoring. Improvements in lSPR can be demonstrated in the case of DNA hybridization [160]. In the case of biological applications, localized surface plasmon resonance, imaging, and microscopy have gained interest. Recent advances in these methods regarding the optical platforms and the functional coatings and directing to the detection of bacterial cells are discussed with respect to many biomolecular interactions such as drug-receptor, protein-protein, protein-DNA, or even protein-cell measurements [17]. The use of portable systems in direct detection of analytes in blood or in diagnostics is advantageous as well as the improvement of the in-coupling of radiation into the metal film. Advantages of compact grating-coupled SPR are demonstrated (GCSPR) [161].

As soon as surface plasmon resonance was accepted as a very good method for measuring concentrations of biosamples, fiber optics was considered as a new miniaturized approach [162]. As an interesting modification, a bifurcated fiber tip coated with a gold film, allowing tip-based surface plasmon resonance. The fiber is dipped, e.g., into the wells of a microtiter plate, and interactions between the tip-immobilized recognition elements and the analytes in the wells are evaluated in the same way as normal SPR. This FO-SPR is commercialized by Fox Biosystems and the approach is comparable with the commercialized biolayer interferometry (see chapter 3.4.3) (http://www.foxbiosystems.com/). The possibility of miniaturization of such fiber-optic SPR systems could be demonstrated [163]. The systems were improved by model numerical calculations, proving experimental results in terms of geometrical structure and materials in the dynamic range [164]. DNA hybridization [165] was validated using a commercial Biacore 3000 system as SPR reference. Using nano-beads in the assay as enhancement, the measurement of allergen could be validated versus ELISA [166]. Determining Alzheimer’s disease via fibrinogen is another application, where the silica core is coated with silver aluminum and nickel [167]. Even pathogens can be detected by combining SPR fiber microdevices with a polymer chain reaction (PCR) chamber [168]. Localized SPR can be used in arrays of vertical gold-SiO_2_-gold dimers, e.g., for a testosterone biosensor [169]. In recent years mainly further optical developments and characterization approaches for improving these miniaturized systems are published in optics journals.

It was even possible to investigate cells [18, 170]. The commercialized Bionavis SPR claims high-quality measurements of surface interactions as well as layer properties and enabling measurement of living cells which is achieved by Multi-Parametric Surface Plasmon Resonance (MP-SPR) (http://www.bionavis.com/en/).

However, since the evanescent field decays within 300 to 400 nm in the bulk, measurement of cells with conventional SPR is problematic. For this reason, long-range SPR has been introduced, where on the glass substrate of the prism a 1299-mm Teflon layer is coated, on which a gold film of only 25 nm is placed [171]. Thus, the penetration depth of the evanescent field is extended to several micrometers. As an imaging system, SPR can be used in cell-based clinical diagnosis [172] or for monitoring dynamics of cell processes using a wavelength-scanning SPR microscope [173]. Imaging LSPR opens the possibility to examine intact cells [174]. Beside the dependence of refractive index on temperature and the decay of the evanescent field into the bulk it has to be considered that in a metal film SPR signal is not localized, but continues for several micrometers. Therefore, for imaging setups, crosstalk between spots or channels may be a problem. It can be solved by localized SPR or by using nanostructures which are functionalized with specific recognition structures for the detection of certain analytes in solution and in combination with so-called GRIN lenses (gradient index lenses) to allow easy optical readout in the far-field modified setup by effects in the near field of the structures. GRIN lenses achieve their focusing properties by spatially varying internal refractive index and image directly the metallic nanostructures as an objective being automatically in focus [19]. Compared with standard microscope objectives, this configuration is more compact and offers advantages in such imaging setups [175]. For imaging systems, the measurement of more than 100 spots in parallel is expected. Thus, most “imaging” systems are in reality just multiplex systems, such as the Bruker SPR and the Sierra SPR-32 system, which enable high-throughput surface plasmon resonance analysis of molecular interactions at 32 individually addressable detection spots (https://www.bruker.com/products/surface-plasmon-resonance/sierra-spr-32/overview.html) or the Biacore 8K as a high-throughput, high-sensitivity SPR system with 8 channels for high throughput and small molecule (https://www.gelifesciences.com/en/us/shop/protein-analysis/spr-label-free-analysis/systems/biacore-8k-p-05540, https://www.gelifesciences.com/en/us/shop/protein-analysis/spr-label-free-analysis/systems/biacore-8k-p-05540#related-documents).

SPR is the most-cited method in direct optical sensing. In recent literature, one interesting communication can be found on smartphone-based SPR [176]. This publication tries to give a status of commercialized SPR biosensor technology, also. At status year 2018, the companies offering SPR instrumentation are named with the designation of sold instruments. Discussion of ultrasensitive SPR [177, 178] is another new topic. An interesting aspect in the monitoring of cell-based assays is the combination of impedance analysis and SPR. Time-resolved measurement of cell adhesion and differentiation become possible [179]. Recently the application of SPR in medical diagnostics is demonstrated [13] and perspectives for small molecule screening are discussed [14]. Mimotopes demonstrate new recognition elements [180]. Their use allows analysis of binding kinetics and interesting perspectives for mycotoxin detection. An extremely sensitive SPR based biosensor, offering increased productivity in fragment drug discovery and measuring small molecules is the commercial Biacore S200 detecting approx. 0.01 pg/mm^2^ (https://www.gelifesciences.com/en/us/shop/protein-analysis/spr-label-free-analysis/systems/biacore-s200-p-05541).

SPR depends mainly on the refractive index. Its changes are influenced by the interaction processes during biochemical reactions. Typically, changes in pg/mm^2^ transducer interface can be detected. However, the refractive index is temperature-dependent. Thus, in addition to the problem of specific discrimination of specific/non-specific interaction, minimal changes in temperature influence the readout of the SPR signal. Accordingly, high-temperature control (< 0.01 K) and/or sophisticated referencing are essential. These problems with the high dependence on temperature applies to all evanescent field techniques and can be called a disadvantage of this type of direct optical sensing. Especially in Homola’s publication examples for this necessary referencing are discussed and solutions given.

Since commercialized SPR supplies software special care has to be taken to know how to properly perform, analyze, and present biosensor data. Understanding of biomolecular processes in the homogeneous phase close to the transducer, transport processes to the surface and kinetics at the recognition site are prerequisites for valid data [2, 116, 181]. Screening more than 1000 biosensor citations the reviewers find that the quality of the biosensor work in these articles is often pretty poor [182]. This review of 2006 could be repeated nowadays with really no improved results.

##### Nanofibers

Optical micro/nanofibers (OMNFs) improve the sensitivity by a large fraction of evanescent fields and high surface field intensity. Using biotin-streptavidin the dependence on fiber diameter is examined and simulated [183]. Compared with former approaches for LSPR and fibers [22, 184] lower limits of detection were achieved. Integrating nanofibers into miniaturized analytical systems promises tools enabling screening, diagnosis, and effective disease management in cancer diagnostic [185].

##### Resonant Mirror

As in the case of SPR with resonance between incident radiation and plasmons in the metal film, waveguide structures without this metal film can demonstrate a resonance like behavior. This approach is the so-called resonant mirror. Radiation incident above critical angle (mostly via a prism) forms an evanescent field at the interface of the high-index substrate to a low index spacer layer. It is coupled into a very thin mono-mode waveguide placed beneath the spacer layer, when the propagation constants in substrate and waveguide match. The waveguide is the resonant cavity. For resonance detection, a reference phase has to be provided. This can be achieved using the TE mode as a reference to the TM mode and vice versa. The resonant cavity of the waveguide will influence the TM and the TE mode differently, and a readout after some distance will change the polarization state of polarized in-coupled light [112, 186, 187]. Real-time analysis was first done with a demonstrator [188]. Binding studies were done with this method using a former instrument by Affinity Sensors Ltd., Cambridge, UK (IAsys 1995) [23]. Prism and grating couplers have been compared [153]. A review of biochemical sensors based on Resonant Mirror is given in [24].

#### Structured fibers and waveguides

##### Structured fibers and waveguides with internal gradient

These devices can be realised as Bragg gratings or Chirped Bragg gratings. Bragg gratings in fibers were first considered to be interesting for telecommunications [189]. Gratings with variation of refractive index were embedded into the fibers through optical processes. These variations inside the core of the fiber select a frequency to be reflected inside the fiber. This can be considered as a certain type of resonator, and later was used not only in combination with external interferometers for readout but also using these internal gratings in various setups as internal interferometers. Radiating the fiber with white light, a dip in intensity is formed within the bandwidth of the transmitted radiation, whereas the back-propagating radiation exhibits a single line, but mostly with sidebands. The possible fabrication approaches of such fiber Bragg gratings are exhaustively reviewed in [190]. Apart from the various fabrication techniques, in this paper, a detailed discussion on the form of the reflection spectrum, the coupling conditions, and different approaches for chirp as well as tilted gratings is given. In addition to use in telecommunications, soon measurements of temperature and strain control became interesting. Due to this interest also in new applications, quite a few tutorials and reviews, and even extensive books could be found in literature, covering this new type of fiber optical technology. Mainly considering the interests in Bragg gratings being sensitive to temperature, axial strain, and pressure, the fundamentals are described in [191]. In a book chapter on optical interferometry [192] Bragg gratings are also considered as intrinsic reflectors in the fiber to construct various types of fiber interferometers such as Michelson or Fabry-Perot interferometers. The combinations of Bragg fibers and interferometric readout, the materials, the fabrication and sensing applications for new smart optical fibers systems have been discussed recently in [193]. A classical book on Fiber Bragg gratings covers fabrication, theory, and characterization. It is also obtainable in Google Books [194].

Until 2000, the focus of Bragg grating development was on application in telecommunications, and on temperature and strain measurements. Then, the first application was published using long-period fiber Bragg gratings in immunoassays, especially for the measurement of antibody-antigen interaction. Typically, in these first experiments, the problem of competition between specific and non-specific interaction was not examined in detail [195]. Bragg gratings can be combined with surface plasmon polaritons (SPP). Two different approaches are discussed; the first is a Bragg grating fiber with cladding, and around the cladding a thin metal film. The second approach is a capillary, where the wall is coated with a thin metal film, and the glass of the capillary contains Bragg grating. In the title, “biomedical application” is mentioned, but is not discussed in the paper [196]. However, interesting is the realization of waveguide/capillary structure. Some further biochemical applications are mentioned in [111].

The necessity of sophisticated biofunctionalization is demonstrated in [197] where proteins were immobilized via only ionic bonding, combined with avidin/biotin linkage, and, finally, covalent bindings combined with an avidin/biotin linkage. As a probe protein, bovine serum albumin (BSA) was used. This early stage of biosensing demonstrates that large molecules and interactions with extraordinarily high binding constants were used first in optics and physics. The results of small-biomolecule immunosensing with plasmonic optical Bragg grating sensors were compared with results of enzyme-linked immunosorbent assay (ELISA). In this case, a surface plasmon resonance optical fiber biosensor based on tilted fiber Bragg grating technology was used for direct optical detection [198]. More sophisticated surface chemistry was used in the case of the detection of thrombin [25]. Comparable with other assay approaches on the Bragg fiber, (3-aminopropyl)triethoxysilane (APTES) with aptamers was immobilized and allowed good thrombin detection. However, the observed “binding curves” and analytical data did not achieve the quality of other optical biosensors. Instead of fibers, waveguides as silicon photonic biosensors in a slot waveguide are also used [26], where the Bragg gratings are formed with a sidewall structuring on the outside of the waveguide within a microfluidic channel. However, the results are not convincing with regard to biosensor quality.

Chirped Bragg grating fibers could show very interesting properties. Either the periodicity of the refractive index modulation is not constant, but gradually increasing, or in some distance within the fiber core gratings with different grating, constants are embedded. In telecommunications, the selection of different frequencies which could be correlated to different interaction processes on the different grating areas are published, but not applied to real biosensor approaches. A review of this chirped fiber Bragg grating [199] refers only to the measurement of muscular activity associated with peristalsis. The development could be interesting for sensor arrays. About such arrays and the possibility of spatial multiplexing, first publications could be found in 1995 [200]. To increase the sensitivity of fiber Bragg grating sensors when measuring the refractive index, tapered fiber optical interferometer (without cladding) between two fiber grating areas was considered to have high sensitivity. This can be called a fiber Fabri-Perot interferometer, first used as a gas pressure high-temperature sensor [201]. This approach was used to detect biomarkers for breast cancer [27] to calibrate HER2 biomarkers, surface functionalization is improved (APTES, cross-linking glutaraldehyde, immobilization of HER2 antibody, blocking by bovine serum albumin) at minimized non-specific interaction. The lowest detectable concentration is 2 μg/L, whereas the cut-off level is 15 μg/L serum [28]. An interesting approach is the combination of optical and opto-acoustic microscopy to image thin samples to make it more accessible to the biomedical community [202]. In this opto-acousting microscopy, a protein transmission mode phase-shifted fiber Bragg grating interferometry was used. No interesting biosensor applications can be found in the literature regarding this method. However, the original use of fiber Bragg gratings to measure bone deformation under load could be interesting for the elucidation of biomechanics of the bone tissue to understand the mechanism of normal remodeling and repair processes, and also effects in bone metabolic diseases and injuries [203]. Recently, such fiber Bragg gratings were embedded in smart garments to measure body postures at different joint positions [204]. Future interesting applications can be expected in the area of biosensing as in the case of SPR fibers.

#### Waveguides with external periodic structure

An extensive amount of research has been done in the area of reflected diffraction gratings with regard to on-chip optical use, aiming at sensing applications. The conventional prism coupler was experimentally replaced by a grating coupler which in general could be called a resonant waveguide grating [205]. The propagation of the guided waves in a waveguide or fiber has been considered according to the theory of periodic dielectric waveguides [206]. It depends on the refractive index in the environment, but also on periodic variations in the boundary, given, e.g., by groove profiles from edging and depending on angle of incidence or reflected radiation as well as waveguide properties. Based on fundamental considerations, a large number of different realizations have been published in the last decades. Recently, a review has tried to classify, to give recent advances, to show numerical modeling, and to survey fabrication techniques of such generally called resonant waveguide gratings (RWG) [207]. Interesting is an integrated-optical Bragg-reflector using a waveguide with relief grating separated from an effective refractive index–shifting element (a dielectric plate with refractive index). Since a membrane can vary the distance electro-mechanically tuning of the Bragg-wavelength becomes possible [208, 209]. A potential biosensor application has not been considered, yet.

##### Incident radiation and readout perpendicular to structure

In 2002, a modification of a structured waveguide was introduced. It contains a sub-wavelength structured surface (SWS) which creates upon perpendicular illumination with white light a sharp optical resonant reflection at a particular wavelength. It is an unconventional diffractive optical set-up. I can be used as a microarray platform, even at normal microtiter plate size [210]. It is called colorimetric resonant reflection. For biomolecular interaction, detection of the term “BIND” was introduced and tested with a polyelectrolyte multilayer on PEG-biotin surfaces [211, 212]. This method was commercialized by SRU Biosystems, Woburn, Massachusetts [29] as the BIND system and introduced for 96-well BIND microplates with 8-channel optical fiber probe. In 2010, SRU Biosystems announced introduction of BIND® SCANNER for primary and stem cell applications [30]. BIND is not anymore on the market.

##### Photonic crystals

A waveguide with grating can be considered as a simple, one-dimensional “photonic crystal”. The basic idea was to design materials which can be compared with ordinary semiconductor crystals that affect the properties of electrons. This is achieved by using a periodic dielectric structure with a periodicity in the order of a wavelength and forms a photonic bandgap. This is achieved by constructing a crystal consisting of a periodic array of microscopic uniform dielectric sites. Photons can be described in this crystal in terms of band structure. The basic concepts and the photon phenomena which can be achieved are discussed in [213]. A “photonic defect” within the bandgap can be introduced by locally disturbing the periodic structure of the photonic crystal. The result is a defect mode. Radiation resonant with the defect mode can propagate in the photonic crystal, and a relatively sharp peak is readout related to the bandgap. This spectral position depends highly on changes in the local environment around the local defect. Some possible realizations of producing photonic crystals with micro cavities are demonstrated in [111]. This can be realized in a photonic crystal fiber where radiation is guided within a periodic array of microscopic “tubes” running along the entire fiber length. These are described in [214] regarding fabrication techniques and light guidance in the fiber. A first pseudo biosensor application is mentioned using silica based fibers filled with dye-DNA solution and measuring the transmittance [215]. Core microstructured polymer optical fibers can also be used. The difference between water and air filled core are demonstrated [216]. Biochemical sensing is achieved by immobilizing monolayer of poly-L-lysine and double stranded DNA on the sides of the holes of a photonic crystal fiber [31]. In [217] in comparison with Bragg gratings the photonic crystal fiber grating is theoretically treated using coupled-mode theory and numerical simulation to explain effects of refractive index, strain, temperature and biomolecules on top of the fiber. Photonic crystals are used in the study of matches of DNA in FRET applications to discriminate single base-pair mismatches [218].

Instead of fibers surface structures can be fabricated on bulk glass or polymer to form slab waveguides. Such a 2D photonic crystal slab with a thickness of the order of the light wavelength is introduced by [219]. The thickness of the photonic crystal slab is just 0.3 μm, and the internal air rods are 0.3 μm in diameter. On top and at the bottom of this square slab, air is forming a clad, and also air is inside the rods. In total, a microcavity array is achieved. This slab is irradiated from the small side, perpendicular to the rods. A defect is introduced by reducing the center pore diameter. Such a configuration gives rise to a resonance in the bandgap. Any change in the refractive index in these cavities or rods causes a shift in the resonance wavelength which can transmit the system. This is demonstrated for DNA or proteins in the microcavities in [220]. A modern approach to fabricate such photonic crystal structures is given by [221], whereby saw-tooth-like anodization new types of photonic crystal structures can be produced based on nanoporous anodic alumina. This can be used as a very effective biosensing platform. In recent years, a large number of publications about the use of photonic crystal surfaces in biological applications have been published. A recent review is given in [222]. Further applications of nanoporous anodic alumina are given in the chapter on reflectometric interference. The combination of photonic crystals and plasmonic nanostructures can be of interest in the future — this 3D photonic crystal incorporated with plasmonic nanoparticles are discussed as recent advances with future perspectives [223]. The combination of a hexagonal photonic crystal fiber with a dual optofluidic channel based on the SPR effect is proposed for biosensing and food safety [224].

Interestingly, Cunningham started to use the term photonic crystals also for fractured slab waveguides which had been considered simply as one-dimensional gratings. Via a molding process, a grating structure was produced on a transparent polyester sheet. Next, the lower refractive index polymer grating structure was coated with a thin film of high refractive index TiO_2_ to receive the final sensor structure. This structure is cut from the polyester sheet and attached to the bottom of a standard microplate. This was later used in the BIND reader of SRU Biosystems mentioned above [225]. Such a system was used later on to measure cell adhesion molecules, plasma membrane-bound adenine nucleotide translocators and metalloprotease as interesting experiments in neurosciences [226] in different configurations. In recent years, such one-dimensional photonic crystals were used to detect colonies of *E*.coli [32], and for protein biomarker detection in microfluidic cartridges in lab-on-chip setups [227].

##### Input/output grating coupler

Various grating couplers of the Lukosz group were another development and were discussed as another device comparable with Bragg reflectors where transmission or reflectance sensitively depended on the effective refractive index within a fiber. In the case of the input grating coupler, the grating was embedded in the surface of a waveguide and the measured power of the in-coupled mode behind the waveguide is affected by the refractive index of volume near the grating. The first experiments are given in [228] for integrated optical switches and measurements of humidity and gases [229]. Further development of this sensor principle was influenced by the optimization of the embossing of gratings in the inorganic material [230, 231]. Experiences of simple waveguide production caused some improvement of grating couplers fabricated from plastics [232]. Further improvements were achieved using films with Ta_2_O_5_, or even with a polycarbonate TiO_2_ waveguide sensor chip [233].

In parallel, Kunz especially worked on waveguide material and the possibility to modify the grating. Non-uniformity of the waveguides results in a spatially varying thickness of the guiding layer [234]. Accordingly, a grating of the effective refractive index is produced, and in-coupling/out-coupling conditions vary across the grating of the waveguide [235]. This means, the grooves of the grating were not embedded parallel, but more as spatially dependent in distance between the grooves. Kunz called it GREFIN (gradient effective index). A similar effect can be achieved varying the thickness of the waveguide perpendicular to the direction of guided radiation. The necessary goniometer for optimum in-coupling was miniaturized [236]. The aspects of different types of smart planar optical transducer chips were discussed and reviewed for different applications in theory and with experiments, mentioning the use of a “chemical disc” [237]. The theoretical background of integrated optical chips for the label-free sensing have been discussed with respect to evanescent field penetration depth, bulk volume refractometry, thin and thick layer sensing and particle sensing in an overview [238]. Soon the interest was directed to biosensor application. The input grating coupler was used to observe an enzymatic reaction [239] or to measure protein adsorption (human immunoglobulin G, h-IgG) [240, 241]. Finally the implementation of integrated input grating couplers as direct immunosensors is discussed with optical requirements and biochemical experiments [242]. The minimal detectable antibody concentration (rabbit anti-h-IgG) was 2 nM or 350 ng ml-1. In 2000 a patent was filed by Tiefenthaler [243] and Artificial Sensing Instruments (ASI) in Zürich commercialized a BIOS-1 instrument. Via a goniometer set up changes in the in-coupling angle to the grating was monitored. Nowadays the company is present in the internet with the aim to develop chips and instruments for biochemical applications, however, no product is presented [244] anymore.

An interesting alternative to input couplers is demonstrated as an output grating coupler with “reversed” path of radiation. Laser radiation is end-fire coupled into the planar waveguide. At the grating the out-coupled beam is focused on a position-sensitive detector, since the output angle varies with refractive index in the bulk next to the grating [245]. The first results for measuring antigen/antibody interactions are given in [246]. The results are compared for input, output coupler and surface plasmon resonance regarding resolution of the shifts in the resonance curves in dependence on changes in refractive index [247]. The results of the Lukosz group are summarized in [33] discussing the different approaches of couplers and difference interferometers. For input/output couplers, prism couplers, and surface plasmon sensors the minimal observable resolution of refractive index changes are calculated and experimentally determined for anti-h-IgG (dips for SPR normally broader, SPR more sensitive). It is stated that calculated resolution might be too optimistic since effects like scattering, spatial inhomogeneities of chemo-responsive layer and its stability are not considered.

The mechanical restriction of adjusting the in-coupling angle has been overcome by using a reflected mode operation. Convergent or divergent beams, respectively, are irradiated onto the grating. The position of reflected radiation is analyzed with a CCD array (in- and out-coupling) [248]. This approach was used for another interesting application. The analyte gradient across the height within a sample cell in dependence on vertical concentration and determination of an interface between different solvents was determined [249].

Based on the principle of input grating couplers in parallel to the development in the Lukosz group a similar setup was introduced as called optical waveguide light-mode spectroscopy (OWLS) [250]. The incident radiation is diffracted by an optical grating at the surface and starts to propagate via total internal reflection inside the waveguide film at a well-defined incident angle. The phase shift during one internal reflection equals zero, and the guided mode is excited. It generates an evanescent field, penetrating into the bulk. Next to the waveguide, the guided mode excites a sharp peak (could be TM and/or TE mode) which can be readout at the end of the waveguide. An instrument prototype is mentioned in [251]. The specific grating material and the measurement of protein/DNA interactions, lipid bi-layers, and even interaction with cells are reported. The method was commercialized by MicroVacuum Ltd. [252]. This instrument was applied to investigate membrane-bound ion channel activities [253] and the adsorption of charged metal nanoparticles as nanostructured material for bioassays [254]. The adsorption and desorption kinetics of flagellin at various conditions were recently published as an approach to determine orientation and surface coverage [255]. Essential for any biomolecular interaction analysis is the fluid handling, the transport processes and the diffusion to and from the interface. These considerations are essential, especially in case of fluid handling in cell-based assays [256], and are discussed in detail for such instruments. Recently, the instrument has been used as a label-free biosensor in Agro-environmental and food safety [34]. OWLS has been compared with quartz crystal micro balance results for real-time direct detection of probiotic bacteria in fermented dairy products [257]. It is stated that OWLS is superior to QCM. Interesting is the combination of OWLS and electrochemistry to monitor the adsorbed mass of charged molecules and to study the reversibility of a adsorption processes [258].

Another possibility of grating coupler or resonant waveguide grating is used with the *EPIC* system, first commercialized by Corning [37]. In this resonant waveguide grating biosensor, in-coupling and out-coupling is used as discussed for living cell sensing [259], and applied to G protein–coupled receptors (GPCR) [260]. This biochemical detection can be combined with a microfluidic cell and several responses through the activation of protease-activated receptor can be monitored [261]. Thus, this system is discussed to be suitable for high-throughput screening. Potential realizations of parallel biosensing are discussed in [36]. For some years, PerkinElmer offers the *EnSpire* Multi-mode plate reader with Corning EPIC label-free technology [262]. The EnSpire Label-free platform can be combined with traditional measurement technologies such as fluorescence, ultrasensitive luminescence, or even time-resolved fluorescence [263]. A large number of drugs on the market target G protein–coupled receptors (GPCRs). The monitoring of label-free cell-based assays come into focus of research [264]. Some new applications in drug discovery are presented in [265]. In a technical note Perkin Elmer compares the performance of the EnSpire Multimode Plate Reader and the Corning® Epic® System [266].

A novel transducer based on gratings was introduced by coating the surface of a chip by an extremely thin waveguide film of amorphous TiO_2_. This is structured with a sub-micron grating relief which is composed of two superimposed uniformed diffraction gratings of different periodicities. This bidiffractive grating serves as both an input and an output port for coupling and decoupling radiation beams to and from a planar waveguide. The bidiffractive grating forms a frequency spectrum which contains two fundamental spatial harmonics [267]. The operation principle is described in [268], and exhibits high sensitivity whereby two fundamental modes are used and the difference angle of the two decoupled modes is measured interferometrically. The direct thyroid-stimulating hormone (TSH) shows a detection limit of 10^−9^ mol L^−1^. This can be correlated with a surface coverage of 24 pg mm^−2^. In a close cooperation between research, industry, and naval medical research command, a bidiffractive grating biosensor was further developed to allow immunoassays for biological threat agents [269].

For the in situ analysis molecular interaction in biological samples, a new method was introduced, called focal *molography*. It visualizes specific biomolecular interaction in real time. The fundamental approach is explained in [35]. The sensor chip is based on a single-mode optical waveguide with a grating coupler. Molography is a molecular nanotechnology for the examination of molecular interactions. Molecules are detected using holography. With these methods, biospecific interaction of biopolymers with an analyte can be visualized using a microscope. Biomolecules are immobilized on a chip in a refraction structure; interaction with a ligand changes the refractive index of the refractive structure, and a coherent optical element as” mologram” is formed. Holography uses photolithography, molecular self-assembly, and laser optics. A mologram is produced by lithography on a photo-reactive biocompatible polymer layer which is formed by self-organization of a wave-guided layer at high refractive index. The mologram is irradiated by the evanescent field of the propagating laser light within the waveguide. Without analyte, no refraction takes place. However, if the analyte binds to the mologram, one finds a holographic structure of the mologram and a focusing of the radiation into a photodetector array [270]. In an extensive paper, the refined theoretical models and measurements of diffraction-molographic foci are presented. It is claimed that the resolution in real-time binding experiments is comparable with that of the best SPR sensors without the need of temperature stabilization or drift correction. The method even allows the label-free detection of low-molecular weight compounds in an endpoint format [271].

### Optical biosensor using interferometry with fibers or waveguides

In the chapters on structured and non-structured fibers or waveguides, the influence of a biomolecular interaction process on the phase of modes, on resonance conditions, and, e.g., grating constants, has been discussed. In some optical realizations, these were combined with interference effects. These could be called single-pathway interferometers since they did not use a reference arm. Devices such as Bragg fibers demonstrated an internal pattern by interference of multiple reflected radiation, whereas in the case of difference interferometers two modes of polarized radiation were measured. In this chapter, methods are considered, which readout an interference pattern, especially. Some of these methods are compared with the discussed waveguide-based methods in [272] or [112]. Some of the interferometric methods have been reviewed in [111, 126, 273].

#### Single pathway

##### Backscattering

Another class of biosensors is called backscattering interferometry (BI) sensor. A single-wavelength laser is focused on a small sensing area, and a detector analyzes the reflected intensity. An interference pattern is produced at the detector, depending on the sub-wavelength structures on the sensing surface. Backscattering has developed as a label-free detection method in the following fields: (a) measurement of small refractive index changes in fused-silica capillaries, (b) monitoring of biomolecular interaction in microfluidic channels, (c) demonstration of bioreactions on porous silicon sensor surfaces, and (d) application to the so-called biological compact disc.

One of the first applications of backscattering was the measurement of biomolecular interaction on porous silicon-based optical systems. The surface is modified in the pores using biomolecular recognition elements. Incident white light on top and at the bottom of the optical interference layer results in Fabry-Perot fringes as an interference pattern [274]. A more interesting approach was the measurement of backscattering in capillaries. First, a tube of capillary dimensions was examined; it produced an interference pattern irradiated by an unfocused He-Ne laser beam (the curvature of the capillary process produces beams with varying pathlengths). The interference fringes are directly related to the refractive index of the fluid in the tube. Such measurements are considered to achieve very low limits of detection - even zettamols are mentioned. However, it must be taken into account that the sample volume is just 350 pL [275]. This principle was applied to the measurement of refractive indices in packed-capillary high-performance liquid chromatography columns, in nanoscale liquid chromatography [276]. More advanced devices are described in [277] later. One can immobilize on the surface inside fused-silica capillary tubes recognition elements. This allows micro-interferometric backscatter detection [278]. Results for going from capillaries to microfluidic channels are reported for measurements of IgG and calmodulin at very low concentrations (nM) [279]. For binding small molecules, aptamers are considered to be helpful. Accordingly, backscattering was used to find binding constants, and to examine assays for bisphenol-A at nanomol concentrations [38]. Even for liquid crystals the method of backscattering was achieved successfully. The performance of such devices was tested in human serum where glucose was detected in competition with cholesterol, other proteins, and triglycerides (8 μM) [280].

To overcome temperature problems, a compensated backscattering interferometer is introduced. Two adjacent regions of the same multifluidic channel are simultaneously interrogated. The shift of interference pattern along the microfluidic channel from two adjacent regions of the channel are used to increase signal-to-noise ratio (nL cell volume, 1,5 fmol Ca-recoverin) [39]. Backscattering was also applied to achieve Taylor dispersion analysis as a simple and absolute method for the determination of diffusion coefficient and the hydrodynamic radius. Instead of normal UV-Vis detection, the measurement of refractive index is a potential alternative detector [281]. Also, characterization of polysaccharides by Taylor dispersion analysis is reported to achieve a powerful sizing technique for macromolecules between nanometers and microns [282]. A modification of the porous silicon technique is a chip with a stamped pattern which contains a gold particles surface in stripes; BSA is binding to the gold particle, and the micro-patterns of such beads function as a type of refraction grating [283]. This so-called backscattering interferometry in rectangular channels (*BIRC*) is used in nanoscale interferometry [284].

##### Grating coupled interferometry

An interesting combination of waveguide with gratings is given by placing on top of the waveguide 3 gratings, the first for in-coupling, the second after the measurement spot for in-coupled modulated reference light and a third for readout [285]. It can be considered also as a mofification of a grating coupler. Small molecules can be detected like epigallocatechin-gallate (458.3 D) [40]. Interaction of microvesicles with coated fibronectin in [286]. Furthermore membrane vesicles have been examined in detail [41]. This optical princliple is commercialized by Creoptix and called WAVE (https://www.creoptix.com/images/pdf/CreoptixWAVE_Brochure.pdf), which is suitable to detect small molecules and membrane proteines.

##### Spinning disc

The idea of backscattering by nanostructured surfaces was used for a new class of analytical sensors to detect immobilized biomolecules with high speed and high sensitivity by using a spinning-disc interferometer. Gold ridges are evaporated on either a silicon wafer or dielectric mirror disc. Commercially available compact discs consist of tracks of pits with a width of half a micron separated approximately 1.6 μm. Information on this disc is readout by focusing a laser spot onto these pits and absorbing the far-field diffraction. Any biomolecular interaction on these gold ridges causes a phase shift if the interference pattern [287]. The additional big cyclic bands on the disc can also be used for internal reference and for parallel detection of different interaction processes [288]. This special type of microarray on a standard digital versatile disc allows the detection of salmonella (including some serovars with selectivity >96% and campylobacters) [289]. The fabrication of bio-gratings (diffractive gratings of bio-receptors) and their characterization as well as the use of commercially available disc drives is reported in [290]. Such discs can be used to transfer lateral flow strategies for fast bio-sensing at high speed to such rotating discs. The rotation of the discs creates the lateral flow of the target solution. The application of BioCD to POCT in given in [291]. In [292], the approach is tested in a fluorescence assay. However, using backscattering it could be transferred to direct optical detection. For the next years increasing interest in such disks and commercialization can be expected.

##### Bimodal waveguide

For another type of waveguide-based interferometer a single arm is embedded into the waveguide. The waveguide is separated in three parts: Into the first one a coherent source is coupled in; after a certain distance, the guided beam reaches a modal splitter which splits the first guided mode into two transversal modes, the fundamental and the first-order modes (second part), which are propagating until the output of the chip. Such a setup can be used as an immunosensor for rapid diagnosis of bacterial infections [293]. The possibility to use a bimodal waveguide sensor in a point-of-care is discussed in [294]. It meets the requirements of portability and disposability. The possibility to produce a bimodal waveguide interferometer–based refractive index sensor on a low-cost polymer platform is reported with a long list of references in [42]. For the detection of serious hospital diseases such as methicillin-resistant *Staphylococcus aureus* (MRSA) a bimodal interferometer provides a rapid method of identification of pathogens [295].

#### Dual pathway

##### Mach-Zehnder interferometer

Based on experiments of Young with double slits a device was developed 1856 by Jamin [296] using interferometry. Some years later Mach [297] and Zehnder [298] proposed a new type of interferometer which had better light paths than the Michelson interferometer. The free-space optics were substituted by integrating the interferometric structure in planar waveguides, which were byproducts in telecommunication and semiconductor industries, which supplied cheap chips [299]. A first one was realized with two grating couplers, a two-channel flow-through cuvette and two interferograms for measuring immunoreaction. A byproduct of telecommunication was used for a chip where one arm was split in two being parallel and joining the two into the output channel. One arm was exposed to a cuvette, an antigen-antibody interaction is measured [300] and characterized by TM field simulations [301]. Using the grating coupler device, immunoreactions down to concentrations of 10^−11^ M of 40 kDa protein could be measured [43]. The telecommunication chip was further developed [302], and new integrated optical substrates for immunoanalytical applications were developed (calculated 10^−9^ mol L^−1^ pesticide with binding inhibition test) [44].

Based on semiconductor and TiO_2_/SiO_2_ waveguides, one Mach-Zehnder arm was covered with a sensor pad, the second arm with a phase modulator [303]. This phase modulator in the reference arm allows setting the operating point of the interferometer to the point of maximum sensitivity. In the following, many groups worked on the miniaturization and optimization of sensor Mach-Zehnder structures using focusing grating couples [304] or adding a third arm in the interferometric system to resolve refractive index changes of 10^−5^ for affinity experiments [305]. Limits of detection values for simazine immunosensors using an improved integrated optical Mach-Zehnder interferometer were achieved (0.1 μg L^−1^ as LOD) [45]. The chips were pig-tailed for in- and output, the phase modulation was optimized, and intense simulations and experiments with respect to the overall system containing the light source, the sensing unit and the electro-optical phase modulation were performed [306]. Lab-on-a-chip microsystems using standard CMOS compatible processes were used to fabricate integrated Mach-Zehnder interferometers with high surface sensitivity and mono-mode behavior [307].

Different types of interferometers, application of microdisc, ring resonators, surface plasmon resonance, and Bragg gratings are experimentally and theoretically compared [308]. The feasibility of sensing even proteins was also demonstrated [309]. Electro-optical, acousto-optical, thermo-optical or magneto-optical working principles had been introduced for improving the sensitivity of Mach-Zehnder chips. In [310], the periodical wavelength modulation principle is introduced which resolves refractive index changes in a published detection limit of 2 × 10^−7^ RIU. By integrating four asymmetric Mach-Zehnder interferometers in a waveguide structure, low limits of detection (7 × 10^−7^ RIU) and high selectivity of aflatoxine M1 on a miniaturized chip with a size of 1 Cent coin was achieved [311]. DNA hybridization was studied with sensing down to some 100 fM [46]. In recent years, quite a few modifications of the original Mach-Zehnder chips with implementation of phase modulation techniques, improvement of in-coupling and out-coupling have been published. By this means, real-time detection of tuberculosis in human urine samples by using a nanophotonic point-of-care platform [312]. Another interesting approach for an optical microfiber reader based on a Mach-Zehnder interferometer has been published [313]. Some novel integrated plasmo-photonic Mach-Zehnder interferometer structures have been designed and experimentally evaluated recently [314]. The different possible limiting factors for the limit of detection of such devices are examined and discussed [315]. In the past year, Optics Express has published some articles dealing with state-of-the-art Mach-Zehnder interferometers and their improvements. Learning from telecommunications, a coherent phase readout by directional coupler and differential detector detection [316], an optimizing optical attenuation with varying electrical power in the thermos-optic phase shifter on the reference arm of the Mach-Zehnder chip [317] or finally implementing an electro-optic comb sensor with some electronics for the phase modulation [318] were fabricated.

Overall, very good limits of detection are published for Mach-Zehnder chips, sometimes only as a result of simulations and calculating with Maxwell equations the influence on the evanescent field via refractive index in dependence on the structure type of the waveguides. However, especially miniaturized Mach-Zehnder chips are mechanically rather sensitive. Furthermore, real samples in biochemical or medicine samples do not at all reach the theoretical values. A latest application of a broadband Mach-Zehnder interferometer with on-chip spectrum analyzers and mode-filtering components has been published for multiplex diagnostics. A photograph of the reader, a diagram of on-chip components, and the results of the biomolecular interaction in case of C-reactive protein is given [319]. An interesting idea is the migration of the Mach-Zehnder chemical sensor and biosensor to the mid-infrared region (MIR) as has been done in the application of the new device for the detection of the herbicide simazine [320, 321]. By these means, selectivity information of MIR and sensitivity of Mach-Zehnder can be combined.

##### Young interferometer

In parallel, the integrated optical configuration of a Young interferometer has been developed [322]. As in a Mach-Zehnder chip, the beam is splitted by an optical Y branch. However, the two arms are not unified on the chip, but give in a free-space arrangement an interference pattern on a CCD array. Compared with conventional Mach-Zehnder interferometers, which normally only result in one intensity value, a total intensity fringe can be monitored [323]. The Young interferometer can be used for bioreactions, and the limit of detection is 750 fg/mm^2^ for biomolecules on the arm [324]. A multichannel Young interferometer using microfluidics on a chip can be used for monitoring immunoreactions, which reduces the protein mass coverage resolutions to 20 fg/mm^2^ by resolving refractive indices of 6 × 10^−8^ [47]. Stepwise binding kinetics are given for IgG and immobilized to protein G [325]. A more detailed examination of interaction processes in comparison with ELISAs is given in [326]. Of interest is the detection of small molecules and a multianalyte approach to discriminate biomolecules. For the small molecule detection, a molecular imprinted polymer was used as a recognition element, and for the multianalyte biosensing, three different antibodies were immobilized to the chip. Interesting is the disposable polymeric Young interferometer sensor chip [327].

##### Hartman interferometer

In parallel to Mach-Zehnder developments, Hartman filed US patents [328, 329] whereby integrated optics was used. Such a multichannel integrated optical sensor configuration was used for preliminary testing monoclonal antibody for salmonella as a foodborne pathogen [330]. A modified version was then used to measure sensitive immunoassays with human whole blood, using human chorionic gonadotropin as a model system (hCG). Measurements of 0.5 μg/L were possible [331]. As realizations of Mach-Zehnder, Young, or Hartman integrated optics, a large variety of different waveguide-based interferometers were developed, either with Y-branched systems, free-space detection, grating input/output coupling, readout of fringes with a CCD, and finally with phase modulation in one of the interferometer arms.

##### Dual polarization interferometry

Another approach is to place two waveguides on top of each other; a laser illuminates the stacks, passes both waveguides, excites the structure, and diffracts into free space. Since the waveguides are close together within a few 100 μm of the end of the stack, the diffracted wavelength generates the well-known pattern of Young interference fringe in the far field. This setup is called dual polarization interferometer [332]. Such a setup is applied to protein absorption systems [333] with an AnaLight Bio200 instrument from Farfield sensors Ltd. (nowadays not on the market anymore) to study a model protein system such as biotin/streptavidin. Changes in the thickness of the layer were examined [334]. Dual polarization interferometry was also used to characterize structural features of proteins and to identify the binding site of matrilin-A-domain to collagen as well as to measure structural changes induced by the presence of zinc ion [50]. In principle, this interferometric method was used to investigate properties of surface coverage and to determine the thickness of ultrathin adsorbed globular protein layers to surfaces, especially in comparison with neutron reflectivity [335]. Also, polymeric dual-slab waveguide interferometers can be used for probing binding events on the waveguide surface. With dual polarization interferometry, refractive index bound to 10^−5^ RIU and detection of 4 pg/mm^2^ are achieved [49]. For the characterization of thin films, the methods of dual polarization interferometry, ellipsometry, and optical waveguide light-mode spectroscopy are compared. Interesting is the application to inhomogeneous films in [336]. Recently, small molecules were quantified in an immunoassay. Preliminary results have been reported for detecting aflatoxin B1 in direct immunoassay with specific antibodies down to 10 ppt of aflatoxin B1 [48].

Modern strategies of fabrication, the use of well-known techniques in semiconductor processing and the integration of optics in waveguide-based chips make these different interferometric methods to very interesting systems. Interferometry results in low limits of detection. The complex electronic phase modulation and interesting channel structure make the system complex, though certainly be overcome in the next years. Grating coupler, photonic crystals, Young interferometers, and Mach-Zehnder interferometers are compared in brief [337]. RIU values of 10^−7^ RIU and surface coverage in the area of pg mm^−2^ are reported.

### Biosensors monitoring interferometric effects in thin layers

#### Ellipsometry

The optical method of ellipsometry allows the determination of physical thickness and the refractive index of a thin layer independently. It is predominantly used to categorize semiconductor wafers as basic material to produce any kind of electronic equipment. The increasing interest in quality control of such wafer material has made ellipsometry instruments more available. Two modes of polarized light are incident on the thin layer surface, and are reflected after multiple reflections within the thin layer. In dependence on the wavelength, the ratio of the resulting amplitudes of the modes and their phase difference give two experimental “spectra”. To these data, a model is fitted which provides the refractive index and the physical thickness of the layer [338, 339]. The application of spectro-ellipsometry to biofilms started with [340]. By these means, the form of biomembranes could be examined and it was possible to differentiate between liposome membranes forming lipid biolayers or micelles. Recently, the ellipsometry of functional organic surfaces and films has been reviewed, either with respect to adsorption of proteins to solid surfaces [341] or discussing the theoretical and experimental concepts and their limitations to achieve even for anisotropic layers the shape of surface structure at nm-scale [342]. Many years ago, biosensors based on imaging ellipsometry were introduced [53]. Protein patterns were achieved. The characterization of immobilization of biomolecules at interfaces to achieve optimum biosensor systems for simple immunoassays [343] came into focus. The imaging technique is discussed in [54] in detail. Applications to a fast and sensitive approach in biosensing can be found in [51]. As an example the label-free detection of hepatitis B virus [52] is reported.

Normally, external reflection is used in ellipsometry. It can be combined with surface plasmon enhancing layers by using a thin metal film between the biolayer and the prism. By this means, ellipsometry is used to achieve high-performance phase-sensitive SPR [344]. Ellipsometry can also be considered as a novel technique to study solid-supported lipid model systems and, e.g., to decode the effect of anti-cancer agents on lipids. This is demonstrated with erufosine which is known as a membrane-acting anti-tumor agent [345]. Using internal instead of external reflection in normal ellipsometry can be of interest. This approach has been reviewed recently, summarizing the principle, the equipment setup, and the current applications of this *total internal reflection imaging ellipsometry* (TIRIE) [346]. Accordingly, this method can be combined with localized surface plasmon resonance (LSPR) in form of nanostructured gold films for the detection of aflatoxin B1 and M1 in direct assays with specific aptamers [347]. Of interest is a recent application of ellipsometry to control the growing of a grafted polymer film during amplification by polymerization for the human genomic DNA detection without PCR since male and female samples could be quantitatively distinguished [348]. Overall, ellipsometry can be a tool used in measuring biomolecular interaction. However, even nowadays ellipsometry is a complex method which requires theoretical understanding and a fit of model to experimental data. Especially at very thin layers the model does not give a final physical answer since in the mathematical solution, refractive index and physical thickness have a strong correlation. For this reason, ellipsometry is preferably used to characterize biosurfaces and is not yet a tool in normal biosensing, and is influenced by temperature effects.

#### Reflectometric interference spectroscopy

In ellipsometry, both polarization states of radiation are used to determine refractive index and physical thickness of a thin layer, normally, incident at a certain angle to the surface. A more simple and robust approach is just measuring reflectance, as introduced by Fabry and Perot [349], of two superimposed beams being reflected at two interfaces of thin layer. At the beginning, this method was used to measure temperature or the thickness of thin layers until in 1912 Buisson and Fabry presented interference measurements on the width of spectral lines of rare earth gases. They compared their own interferometer with other multiple beam instruments. A long time after, this simple method of interferometry was compared with, e.g., Mach-Zehnder interferometer, and was used for wavelength control and even wavelength stabilization. The first use of Fabry-Perot interferometry was to determine refractive index in micro cuvettes (pathlength 100 μm). This was demonstrated as direct optical detector in HPLC measurements [121] in wavelength regions where analytes do not absorb and show just refractive indices like sugars. Only a few years later, interferometry at thin layers was introduced to chemical and biochemical sensing [350]. Theory and experimental data are discussed for this type of white light interference with multiple reflections in combination with a flow injection analysis setup [351] and named *Reflectometric Interference Spectroscopy* (RIfS). The incident and reflected radiation is along the optical axis to wimplify optics. The shift of the interference spectrum with varying physical thickness of the layer was used to monitor interactions either with gases or with biomolecules. Binding curves could be determined since the method allowed simple time-resolved measurements even without any kind of thermostating. The optimization of the layer system with respect to relative refractive indices of the biomolecular recognition elements, the bulk, the transducer, and the thin interface were discussed [352]. Signal was enhanced coating to the glass transducer an interference layer of a few nm of Ta_2_O_5_ on top of 500 nm of SiO_2_. By this means, good results for atrazine could be achieved using a binding inhibition assay and resolvable changes of less than 3 pm [353]. RIfS not only allows in interaction measurements to obtain equilibrium values for thermodynamics, but also rate constants for kinetics. A quantification of even small molecules is possible for high equilibrium canstants [55]. This is demonstrated for the determination of association and dissociation rate constants as well as for the equilibrium constant for a number of phosphate diester oligonucleotides to determine mismatches. Thus, possibilities are found to improve metabolic stability and pharmacokinetic properties, and to increase the affinity of the antisense/sense interaction which is interesting for multidrug resistance [354]. RIfS was also demonstrated as simple method to detect submonolayer coverage of untagged DNA oligonucleotides [355]. The determination of affinity constants of oligonucleotide duplex formation is relevant for the understanding of hybridization. Nuclease stable double stranded oligomers and high affinity can be created by locked nucleic acid (LNA). RIfS allows the determination affinity constants for the different DNA/DNA and DNA/L-DNA strands [356].

In environmental analysis, limits of detection below 1 μg/L have to be achieved. This is normally done by using labeled reagents. Nevertheless, really good antibodes allow even measurements with direct optical methods such as reported in [357] where benzo[α]pyrene was determined at LOD of around 1 μg/L. A binding inhibition assay was used and optimized by determining thermodynamic and kinetic constants of ligand recognition interactions. Another complex matrix in environmental or food samples is milk which has gained increasing importance in the last years. The problem is non-specific interaction caused by the components of milk which can reduce the quality of direction optical sensing approaches. However, it was possible to establish an assay for the hormone testosterone, with limits of quantification of 130 ng/L [56]. In aggressive matrices, antibodies cannot be used as a recognition element. Instead, molecular imprinted polymers (MIPs) have been considered as good recognition elements. However, good recognition not only at surface but also in volume (larger number of recognition sites) requires a rigid polymer for keeping selectivity. Accordingly, response times are drastically increased. Good accessibility in volume requires non-rigid polymer with loss of selectivity. To overcome this problem, molecular imprinted nanospheres have been introduced as an approach for a robust and label-free detection of small molecules. This is especially essential in fermentation control, not looking for pH, temperature or CO_2_, but rather for controlling the expected fermentation product. This was demonstrated for phenylalanine alanide [358] or for monitoring penicillin G production which is a typical system in fermentation [359]. Further applications are the quantification of sarcosine [360], the determination of myoglobin at 0.1 mg L-1 by immobilizing the antibody via histidine-tagged recombinant protein A [361], or the analysis of biological toxins and *E. coli* ([362]. By optimizing the layer system C-reactive protein was measured [363].

Plaque accumulation, leading to inflammatory processes and failure of dental implants, depends on pellicle formation on which salivary proteins in adsorption and disassociation processes were monitored by time-resolved reflectometric interference spectroscopy. Two established promising candidates for biological coatings of titanium dental implants [364] could be used as a sensor system. Biodegradation of thin films is of interest. Accordingly, polymeric thin films are examined [365]. Interesting is to enlarge the thickness of the effective substrate by different sphere diameters of nanoparticles. Modulation is increased by this colloidal film, and the result is demonstrated by monitoring the digestive process of gelatin by trypsin [366].

Another complex matrix is blood or serum. Instead of recording a spectrum and calculating the concentration from the shift by biomolecular interaction, the setup can be simplified using a LED selected for optimum wavelength dependent on the layer system. With such instruments, *Salmonella* can be quantified in a direct assay at a limit of detection of 1.2 mg L^−1^ [367]. Cystatin C is an improved marker for renal failure. A binding inhibition assay allows the determination of 50 μg L^−1^, which well covers the relevant clinical range [368] Depressive disorders are of global interest. For this reason, as a model system, the drug amitriptyline was measured in human serum (LOD 540 ng L^−1^) at levels which are interesting for therapeutic concentration ranges [369].

Another possibility to increase the signal modulation is the distribution of pores with a different size and depth of porous silica SI chip and use of Fabry-Perot reflectance fringes caused by the reflectance at the top surface and at the bottom of the pores. Accordingly, such porous surfaces result in a 0.1 pg mm^2^ resolution [57]. Such nanoporous anodic alumina (NAA) surfaces with high modulation of the Fabry-Perot reflectometric interference spectroscopy are especially proposed by the group of Losic [370]. This approach is also used for functionalization of nanoporous silicon and microporous silicon with different silane or polyethylene glycol derivatives, and filling the pores with collagen or BSA as linkers or proteins. This opens possibilities in drug delivery and cell biology [371]. In case the anodization profile is modified and forms within the pore structures, distributed Bragg reflectors for the selective detection of vitamin C molecules in combination with RIfS can be discussed [372]. With the same concept, the binding affinity between human serum albumin (HAS) and indomethacin molecules can be monitored time-resolved with RIfS. Modification of the pores allows the establishment of binding affinity between a set of drugs such as indomethacin, cumarin, sulfadimethoxine, warfarin, and salicylic acid [373]. Another possibility the formation of ZnO_2_-based hybrid thin films and mesoporous silica coating all on a glass substrate. It allows the measurement by RIfS the concentration of organic compounds down to a few parts per million [374]. Approaches to engineer the surface chemistry for interferometric sensing platforms based on this nanoporous anodic alumina is given in [375] whereby RIfS is monitoring in real time. Protein-modified nanoporous anodic alumina platforms can be finally used to observe binding interactions of blood proteins. An example is an assessment of the binding affinity between Hg and transferrin-functionalized pores [375]. Finally, streptavidin-biotin complexes are used to measure thrombin [376]. The innovative strategy for optimizing hierarchical structures of NAA is reviewed and evaluated as interferometric bilayer [377]. This allows the fabrication of a variety of functionalized systems with sophisticated multilayer structures within the pores and the possibility to monitor ions [378]. Certainly, an increase in selectivity can be achieved by these porous structures since their thick layer increases the interaction sites resulting in higher modulation signals. However, approaching layer thicknesses in the area of micrometer, the increased modulation frequency requires a good wavelength resolution to get the necessary resolution for calculating the shift by the interaction process from the spectrum shift.

Whereas SPR can be easily combined with MS in a MALDI-TOF mass spectrometric arrangement where the gold layer supports laser desorption, such an approach is considered more difficult for RIfS. However, using an ITO-coated (indium tin oxide) glass support allows the combination with mass spectrometers. This could be demonstrated for the determination of quantitative and qualitative binding processes of mixtures of vancomycin derivatives [379]. In contrast to SPR, with RIfS, there are no problems to combine reflectometry with electrophoretic flow conditions. Thus, it was possible to use RIfS for the detection of relevant biomolecular interactions under electrophoretic flow conditions. This is demonstrated for an electropherogram of a DNA sample obtained using a functionalized LNA (locked nuclear acid) surface with a RIfS detector [380]. Furthermore, RIfS can be combined with fluorescence measurement. An example is the study of ligand-receptor interactions in the plane of membranes in the case of IFNα2 (cytokine human interferon alpha-2) with the extracellular domains of its receptor subunits ifnar1-EC [381].

The capability of measuring cell adhesion using RIfS is demonstrated in [58]. The simple optical setup is advantageous compared with other interferometer systems and is by these multiple reflections very useful for measuring vertically independent even large molecules or cells or membranes. Unlike in evanescent field detection methods, the interaction between radiation and matter is independent of radiation propagation [382]. Reflectometric interference spectroscopy is combined with quartz micro balance to have a useful tool to quantitatively analyze molecular adsorption of vesicles on various surfaces [59].

Endocrine-disrupting chemicals (EDCs) can potentially interfere with the human hormone system. One of the main targets of EDCs is the nuclear receptor superfamily of proteins playing a crucial role in the human hormone system. The question is whether a developed biosensor not only allows the differentiation between ligands and non-ligands of a receptor but also can determine the potential of these ligands to influence conformational changes in the receptor. In consequence, this may lead to activation or inhibition of receptor-dependent pathways. Now, EDCs will interact with these receptors in a different way. Measuring the concentrations of the different EDCs does not give information about their effect on the receptor. One has to determine the dose. This can be called effect-based analytics, and has found interest in recent years. It could be demonstrated that the RIfS is able to discriminate between agonistic and antagonistic effects of potential ligands [383, 384]. A disadvantage of measurements in the UV/Vis region is the lack in selectivity. However, in the mid-infrared wavelength range, a sensor provides additional information via weak absorption spectra (fingerprints). Originally poor spectra can be magnified by surface-enhanced infrared absorption (SEIRA). Thus, the transfer of RIfS from the visible to the mid-infrared could be of advantage [385, 386].

#### Biolayer interferometry

In the case of RIfS via fiber optics, radiation is sent to a surface and reflected radiation is collected and guided to a detector (camera). This allows flow injection analysis with a permanent flow passing the cell on top of the transducer with defined conditions for association and dissociation kinetics. In contrast, in the so-called biolayer interferometry (BLI), the tip of the fiber is modified with recognition elements, dips into solution, and measures RIfS at the tip of the fiber. The principle has been commercialized as Octet from Fortebio where parallelization is achieved by using a microtiter plate in which an array of fibers is dipping in. As an advantage is given that no fluidics is necessary and “homogenization” is achieved by the so-called orbital flow-through shaking of the microtiter plate. Sometimes it is mentioned that by this type of fluid control, the dissociation rate constants can be influenced, and it must be considered that more mechanics is necessary to lower the fibers correctly into the wells [66]. Furthermore, problems by blocking of the needles is discussed. This setup allows array-based measurements as has been performed in epitope discrimination for the selection of monoclonal antibodies with functional activity [387]. BLI can also be useful to predict an ELISA performance of antibody pairs when an antigen contains many repeats in its sequence. This can be of interest for many targets using BLI as a predictive tool [63]. The possibility to use a commercial instrument results in many applications such as the quantification of monoclonal antibody cell culture titer, providing a fast and cost-effective alternative assay in cell culture harvest [388]. By using Langmuir Blodgett methods, uniform large-area 2-D materials are achieved, and these films can be used for biolayer interferometry. The detection of specific binding of pathogen-derived proteins is reported [67]. BLI also allows the characterization of biofilms in real time which is usually a challenge. Thereby, the initial rate and the final biofilm deposition on surfaces can be examined to find out the effect of antibiotics such as carbenicillin, tetracycline, chloramphenicol, and colistin. These antibiotics readily cause the formation of filamentous bacteria at concentrations both above and below their minimum inhibitory concentration (MIC) of an antimicrobial [64]. *Pseudomona aeruginosa* efficiently adheres to human tissues, including the lungs and skin. This causes infections that are difficult to treat. A main component of the extracellular matrix is laminin, and the specifics of interaction could be confirmed using BLI. In a recent scientific report, the consequences are discussed based on the BLI obtained data [65].

Another new type of label-free biosensor uses spectral correlation methods which correlate signals between two coupled interferometers — it is called a picoscope. One of the interferometers is the glass slide with the recognition spots or wells, the second one is a scanning interferometer that employs periodical modulation of path differences of the interfering beams [389]. The quality of expoxilated or biotinylated sensor chips used in this type of spectro-correlation interferometry is reported with LOD values in the area of mg/L [390].

### Imaging

Rather early in the area of direct optical screening started approaches to parallelize transducers. In view of drug screening, selection of alternative binders out of cell cultures, and personalized medicine imaging methods are of high interest. Imaging is available in resonance and interferometric methods. Boundary conditions are necessary parallelization, crosstalk between spots, and achievable lateral optical resolution. One of the first attempts was to use a liquid crystal-based Lot type filter which selects one wavelength after the other incident in parallel for 40 wells for RIfS. For better Signal/Noise ratio the sampling time of the reflected radiation was 30 ms. Within 300 s, about 20 thickness values for reflectometric interference for each well could be recorded giving the binding curves [391]. For a prototype, the Lyot filter was substituted by a filter wheel which selected 7 wavelengths out of the white light. Screening experiments were done selecting thrombin inhibitors in a 96-microtiter plate. This setup can be called the start of imaging technology for RIfS [392].

A SRIB system (spectral reflectance imaging biosensor) of another group uses a little different approach. A laser as light source irradiates via a beam splitter the glass slide and the reflected radiation of hundreds of spots is monitored with a CCD. Surface-bound concentrations and masses of adsorbed layers of ssDNA, BSA, and IgG are measured [393, 394]. This system was renamed to IRIS (interferometric reflectance imaging sensor), and instead of the laser, 3 LEDs were used to get the interferometric information. A comparable sensitivity as for the Biacore system is reported for protein-protein, DNA-DNA, or antibody-antigen interactions [60]. Instead of few wavelengths as in the single spot RIfS without beam splitter, just one wavelength supplies enough information also in imaging as long this wavelength is optimized to the transducer system. The quality was tested in a setup with flow cell and tubing pump using a laser and a CCD. It is called pi-RIfS, since polarized light was tested. In a 5 × 5 microarray the antigen-antibody interactions relevant for diagnosis of the antiphospholipid syndrome as an autoimmune disease were successfully tested [61]. The mentioned imaging instruments will allow the realization of versatile tools for fragment-based screening and the future drug design process [395].

In the next step, 1-Lambda RIfS (iRIfS), based on the pi-RIfS system, was used with a flow cell with 50 μL which allows reagent volumes of less than 300 μL. Since polarized radiation did not show advantages, a laser or a simple LED as light source was used. More than 10,000 spots at sizes of 200 pixels (spot diameter approx. 100 μm) can be monitored. Multistep assays were used to demonstrate feasibility [62]. The iRIf and a modified prototype called SCORE were used as a platform for copying microarrays [396, 397]. This system was also applied to multiplexed antibody detection from blood sera by immobilization of in vitro expressed antigens [398].

SPR imaging (SPRi) is a straight forward format for two-dimensional (2D) array sensing which started in 1988 with surface plasmon microscopy [20]. In the literature, one finds SPRi based on reflectivity, angle, wavelength, phase, or polarization interrogation. These different techniques are discussed in detail in [399]. An application is the measurement of whole cells using the specific binding of cell surface antigens expressed on the surface of cancer cells and specific ligands deposited on a sensor chip in a label-free approach using an IBIS MX96 SPR imager [21]. This instrument (https://www.ibis-spr.nl/product/ibis-mx96/) is based on angle-resolved SPRi as developed by Beusink [400]. Further development goes to Microscope Objective-Based, Nanoparticle/Nanostructure-Based, and Smartphone-Based SPRi. The novel use of resolution-optimized prism-based surface plasmon resonance imaging (RO-SPRI) and data processing is described for the detection of the foodborne pathogens *Listeria monocytogenes* and *Listeria innocua* [401]. This paper documents the complexity of modern imaging. Other SPR multiplexing instrumentation has been mentioned in Chapter 3.2.1.4 (https://www.bruker.com/products/surface-plasmon-resonance/sierra-spr-32/overview.html, https://www.gelifesciences.com/en/us/shop/protein-analysis/spr-label-free-analysis/systems/biacore-8k-p-05540, https://www.gelifesciences.com/en/us/shop/protein-analysis/spr-label-free-analysis/systems/biacore-8k-p-05540#related-documents).

### Detection methods reviewed and compared

In the previous chapters in this paper, reviews have been cited which discuss developments of basic optical detection techniques and group detection methods or compare specifications. They provide detailed information about the underlying physics and optics of these methods and discuss applications. These are listed and classified in this review together with some interesting technical reports comparing instrumental parameters of experimental models in labs, prototypes or commercial instruments.

These reviews started two decades ago. Many of the optical probes and transducers were compared with respect to optical effects, substrate materials, spatial resolution, and interaction areas. Optics was assessed in comparison with biochemical and surface properties, diffusion control, interaction kinetics, and fluidic requirements [402]. Reflectometric interference, SPR and resonant mirror (IAsys) were compared regarding the determination of rate constants and binding curves [403]. For grating couplers (ASI, Zürich), interferometric biosensors (Young interferometer IBS 201, Freiburg), and reflectometric interference spectroscopy (BIAffinity, Analytik Jena), the measurements of clinical samples are compared [404]. A first review on direct optical detection methods was published [405] and updated [406]. For integrated optical sensor platforms, the refractive index units are compared [127]. Strategies for label-free optical detection are given in detail in [407]. Development and applications of input/output couplers have been reviewed [408]. Plasmon-enhanced optical sensors are discussed in comparison with surface-enhanced Raman approaches [409]. Similar discussions with added interferometry can be found in [114]. More general reviews deal with SPR, interferometers, waveguide-based sensors, ring resonators, photonic crystals, and Bragg gratings [111]. A similar field of optical methods (interferometers, waveguide sensors, and grating couplers) is reviewed in detail in [126]. Another recent review covers SPR, grating couplers, photonic crystals, ring resonators, and interferometers [410]. Fabry-Perot interferometric fiber-optic sensors are discussed with applications in [411]. These are compared with Mach-Zehnder and Bragg grating [412]. SPR, optical waveguide light-mode spectroscopy, and dual polarization interferometry are discussed with respect to characterization of peptide binding, membrane-mediated events and kinetic analysis of binding mechanism [413]. Now, in the present review, both refractometric and reflectometric realizations of transduction are handled in their entirety.

Recently, two studies of routinely used biosensor platforms have been published. In the first one, the determination of high-affinity antibody-antigen binding kinetics has been evaluated in detail. The authors used a Biacore T100, the ProteOn XPR36, the Octet RED384, and the IBIS MX96. Three of these are based on SPR technology; the Octet uses BLI. All these instruments have their advantages and disadvantages regarding throughput, consistency of data with high quality, and fluidics. Details are given in [414]; company links are provided in [415]. The second study compares Biacore 3000, Octet RED96, IBIS MX96, and 2 imaging RIfS instruments [416]. Some instruments allow multiplex measurements (less than 100 spots in parallel), only for the imaging iRIfS more than 1000 spots can be measured in parallel. Typical limits of detection, throughput, and amount of necessary reagents are listed. In both studies, the best choice depends on the analytical problem, and shows advantages or disadvantages for SPR, BLI, and iRIfS regarding throughput, necessary chip quality and material, association/dissociation kinetics, necessary sample preparation, and temperature control.

## Conclusion

Most of the cited publications provide data such as limit of detection or minimal detectable material coverage on the transducer. However, sometimes they are given as detectable Mol (number of particles), as detectable mass (without providing area on the transducer), or correctly in mol/L as a concentration value. The latter allows a good comparison and can be discussed independently of the optical method, application problem, recognition elements (loading of the surface) or matrix (problematic blood, milk) used. Especially in presentations of new devices (with mostly just marginal differences to the original standard optical concept) the authors present either very small arbitrary units of refractive index or the results of theoretical simulations using the optical parameters. Published very small LOD values can be the result of very small sample cell volumes, e.g., a few picoliters. Measurements with gases (with estimation of values in case of biomolecular interactions) or “model measurements” using the biotin/avidin interaction are used to prove this “new” method. An attempt is made in Table 1 to list at least published specifications for the different detection methods regarding the possibility to detect small molecules or cells, given limits of detection in various matrices, and the capability of multiplexing or imaging.

**Table 1:** Comparison of different direct optical biosensors and applications

	Small molecules	LOD/Matrix	Cells	Imaging/multiplex
Fiber- or waveguide-based				
Coated fiber	–	Gas, flow rate [6], protein A, acetylcholine 5 10^−3^ M [7], μRNA 10 fM [8]	–	–
Ring resonator	–	Biomarks in blood 1 μg/L [9], pM–nM [10]	–	–
Difference interferometer	–	Atrazine 25 mg/L [11], IgG 5 × 10^−9^ g/mm^2^[12]	–	–
SPR	[13, 14],	Biomarkers 500 ng/L [15], nucleic acids fM [16],	Bacteria, protein cells [17–19]	Microscopy [20], multiplex (https://www.bruker.com/products/surface-plasmon-resonance/sierra-spr-32/overview.html, https://www.gelifesciences.com/en/us/shop/protein-analysis/spr-label-free-analysis/systems/biacore-8k-p-05540, https://www.gelifesciences.com/en/us/shop/protein-analysis/spr-label-free-analysis/systems/biacore-8k-p-05540#related-documents), cell imaging [21]
Nanofibers	–	Nanoparticles biotin-Streptavidin [22], cancer 4 × 10^−5^ μg/L	–	–
Resonant mirror	–	Rowth factor receptor 0.1 nM [23], DNA 77 nM [24]	–	–
Bragg grating	–	Thrombin 0.5 nM [25], 2.4 × 10^−4^RIU [26]		
Chirped Bragg grating	–	HER2 biomarker 2 μg/L serum [27, 28]	–	–
Sub-wavelength structured surface SWS	Warfarin [29]	1 pg/mm^2^ [29]	Protein-cell interaction [29]	BIND SCANNER [30]
Photonic crystals		Lysin-DNA 10^−4^ RIU [31]	*E. coli* [32]	
Input/output coupler		IgG 50 μg/L [33]		
Optical waveguide light-mode spectroscopy		Trifluralin 2 × 10^—4^ ng/L [34], bidiffractive for TSH [35], molography 100 fg/mm^−2^	GPCR [36]	EnSpire Multimode Plate Reader [37]
Interferometry				
Backscattering	Norepinephrine in serum 1 μg/L [38]	Ca^2+^ 60 nM [39]		
Grating-coupled interferometry	Creotix wave 0.01 pg mm^−2^ (https://www.creoptix.com/images/pdf/CreoptixWAVE_Brochure.pdf)	Epigallocatechin-gallate 5 pg/mm^2^ [40]	Membrane vesicles [41, https://www.creoptix.com/images/pdf/CreoptixWAVE_Brochure.pdf]	
Mach-Zehnder interferometer	+	40 kDa 10^−11^ M [43], pesticide 10^−9^ mol/L [44], simazine 0.1 μg/L [45], DNA fM [46]		
Young interferometer		Affinity reaction 750 fg/mm^2^ [324], refractive index 6 × 10^−8^		4 channel [47]
Dual polarization interferometer	Aflatoxin 0.01 μg/L [48]	IgG 4 pg/mm^2^ [49],	Matrilin-A-domain to collagen [50]	
Ellipsometry		IgG 15 μg/L [51]	Hepatitis B [52]	BIA [53, 54]
Reflectometric interference spectroscopy	Biotin 40 nM [55]	Testosterone 130 ng/L [56], protein 1 pg/mm^−2^ [57],	Cell adhesion [58], layer, vesicles [59],	DNA 5.2 pg/mm^2^ [60], antiphospholipid syndrome [61], peptide libraries [62]
Biolayer interferometry		HRP2 [63]	Cell wall–targeting [64], *Pseudomona aeruginosa* [65]	Multiplex [66], Langmuir-Blogett [67]

The quality of the optical detection method is one aspect. However, direct optical detection normally takes place at a surface and results in a heterogeneous assay. Thus, the other aspect of biochemical assay has at least the same importance. As mentioned for various applications, the quality of the biolayer, the affinity of the recognition element, the equilibrium constant of the interaction recognition element/analyte, the size of analyte, the viscosity of the matrix, the loading of the surface with recognition elements, the transport process of the analyte to the surface-immobilized recognition element (rate-determining step in consecutive reaction transport to surface/binding kinetics at surface), the quality of the shielding layer to reduce/avoid non-specific interaction, the selectivity, backbonding, and microfluidics (flow rate, transversal homogeneity) influence the observable signal. The assessment of all these dependencies are worth being covered in detail beyond this review on direct optical detection methods in a further critical review. Some of these dependencies have been discussed in previous papers [3, 117, 181, 182, 417].

Commercial biosensors platforms provide software for the evaluation of data and for the determination of kinetic constants which can be used as a black box. Misuse can result in wrong results. For a correct evaluation, the conditions as discussed above and proposed kinetic formalism must be considered [417]. Accordingly, software as provided by the commercial instruments, and by Scrubber (http://www.biologic.com.au/scrubber.html) or Anabel [418], can be used only if the biomolecular interaction conditions are understood.

Thus, most direct optical methods allow to quantify surface loadings of about 1 pg/mm^2^ or, by plasmonic enhancement, perhaps 2 orders of magnitude less. Accordingly, values of 0.01 μg/L in solution for normal small organic molecules can be expected. Table 1 tries to summarize the different types of forms for the limit of detection. Since generally the information on biochemical conditions is insufficient in most papers, a calculation to either quantifiable concentration in mol/L or mass pro detection area g/mm^2^ is difficult. To enforce that such information is provided should be an objective of the biosensor community. The increasing interest to measure cell or membrane interactions cause problems in case of refractometric methods, since the evanescent field decays, even in case of LRSPR. In the case of multianalyte measurements or high-throughput screening, crosstalk between different spots, as it occurs in refractometry by plasmonic effects or propagation in waveguides, must be avoided.

In general, the direct optical detection methods measure the product refractive index *n* times the physical thickness *d* at the heterogeneous phase at the transducer. Both depend on temperature inversely. Evanescent field methods predominantly measure the refractive index and its changes. Thus, they essentially need extreme referencing of temperature influence. Reflectometric methods which measure the superposition of the reflected beams at the boundaries of the biological interlayer and its changes have no problems to detect even cells (no decay as for evanescent fields) and show negligible temperature dependence (decrease of refractive index with temperature is compensated by increase of layer thickness). Reflectometric interference is advantageous in measuring large-size analytes and with respect to temperature control.

Overall, it can be said that in principle most of the direct optical methods end in the same optophysical range. The application and the matrix of the sample determine the selection of the optimum optical method, the biomolecular interaction process, and the necessary assay format. These parameters govern the bioanalytical quality.

This review wants to point out that for many applications numerous slight modifications of the basic optical principles claiming optimisation canbe found in the literature. Therefore, each upcoming “new” method has to be critically considered with respect to improvement obtained by more complex optics or biochemistry. At present, for all applications, a direct optical detection method or even some commercial platforms are available, which are competitive to other detection methods. Developments in optics and electronics in the last decade have promoted miniaturization and parallelization. Thus, for many applications direct optical detection is the method of choice.

Such biosensors are part of Analytics 4.0 [419]. They allow better process control, are essential for personalized medicine, will be interesting for citizen science, supply the necessary megadata for artificial intelligence [420], will help to meet challenges in analytical chemistry in the future and will prepare it for the Internet of Things [421]. It will be interesting whether for biosensors the ideas of digital twins in manufacturing or simulation processes can provide better comparable standardized information on results of biomolecular interaction analysis [422] in research and quality control .
